# Gut microbiota modulation in GLP-1RA and SGLT-2i therapy: clinical implications and mechanistic insights in type 2 diabetes

**DOI:** 10.1093/ckj/sfaf351

**Published:** 2025-11-14

**Authors:** Mehmet Kanbay, Rama Al-Shiab, Ermeena Shah, Lasin Ozbek, Mustafa Guldan, Alberto Ortiz, Denis Fouque

**Affiliations:** Department of Internal Medicine, Division of Nephrology, Koc University School of Medicine, Istanbul, Turkey; Department of Medicine, Koc University School of Medicine, Istanbul, Turkey; Department of Medicine, Koc University School of Medicine, Istanbul, Turkey; Department of Medicine, Koc University School of Medicine, Istanbul, Turkey; Department of Medicine, Koc University School of Medicine, Istanbul, Turkey; Department of Nephrology and Hypertension, Health Research Institute-Fundación Jiménez Díaz University Hospital, Universidad Autónoma de Madrid (IIS-FJD, UAM), Madrid, Spain; Department of Nephrology-Nutrition-Dialysis, HCL-Lyon Sud Hospital, Univ. Claude Bernard Lyon, Pierre Bénite, France

**Keywords:** GLP-1 receptor agonists, gut microbiota, SGLT-2 inhibitors, type 2 diabetes

## Abstract

Glucagon-like peptide-1 receptor agonists (GLP-1RAs) and sodium-glucose cotransporter 2 inhibitors (SGLT-2is) have been shown to provide extra-glycemic advantages, such as cardiovascular and renal protection, in the treatment of type 2 diabetic mellitus (T2DM). Recent data points to the possibility that gut microbiota modification may contribute to their beneficial impact. This review examines changes in microbial composition, metabolite synthesis (such as short-chain fatty acids (SCFA), bile acids, and endotoxins), and their systemic implications by integrating clinical and preclinical data on the interactions between various drug types and the gut microbiota. GLP-1RAs may favor certain taxa that synthesize SCFA and *Akkermansia muciniphila*. This may improve insulin sensitivity and lower inflammation. Likewise, SGLT-2is may favor a eubiotic state, which is associated with better renal and metabolic outcomes. We also discuss the use of baseline microbial profiles to predict therapy responses in a microbiota-informed precision medicine approach. Larger human investigations are required to explore causality and therapeutic efficacy, as mechanistic insights are still limited despite early encouraging findings. This narrative review synthesizes both clinical and preclinical data identified through PubMed, Scopus, Web of Science, Embase, and Google Scholar up to May 2025. Personalized holistic T2DM therapy plans that integrate both host and microbial pathways may be made possible by gut microbiota studies.

KEY LEARNING POINTS **What was known:**

- GLP-1 receptor agonists and SGLT-2 inhibitors can modulate the gut microbiota, potentially contributing to their cardiovascular and renal protective effects beyond glucose lowering.
- Increased production of beneficial microbial metabolites, such as short-chain fatty acids, and reduced levels of harmful endotoxins, such as lipopolysaccharides, are associated with improved metabolic and inflammatory profiles.

**This study adds:**

- GLP-1 receptor agonists, including liraglutide and semaglutide, enrich beneficial bacteria such as *Akkermansia muciniphila*, and help maintain gut-barrier integrity and immune regulation.

**Potential impact:**

- SGLT-2 inhibitors, such as dapagliflozin and empagliflozin, promote a more balanced microbial ecosystem and reduce levels of gut-derived uremic toxins linked to kidney disease progression.
- Baseline gut microbiota composition may influence individual responses to GLP-1 receptor agonists and SGLT-2 inhibitors, supporting the potential for microbiota-informed personalized treatment strategies in type 2 diabetes.

## INTRODUCTION

Type 2 diabetes mellitus (T2DM) is the leading cause of death worldwide, affecting >500 million people. It has serious complications such as cardiovascular and chronic kidney disease. Recent developments in the treatment of diabetes have gone beyond glycemic control with the approval of glucagon-like peptide-1 receptor agonists (GLP-1RAs) and sodium-glucose cotransporter 2 inhibitors (SGLT-2i), both classes with significant cardiovascular and renal protective effects demonstrated in large randomized controlled trials [1, 2]. These therapeutic agents have established clinical benefits and are currently widely used. However, the underlying nephroprotective mechanisms, particularly those involving indirect pathways such as gut microbiota modulation, remain to be fully elucidated.

The gut microbiome has increasingly been recognized as one of the most important components of metabolic regulation and disease progression. Emerging evidence supports the idea that the therapeutic effects of GLP-1RAs and SGLT-2i may be mediated, in part, by the modulation of gut microbial composition and function. In particular, changes in specific microbial taxa and related metabolites may be associated with improved metabolism, inflammation, and vascular markers [3].

This review examines whether modulation of gut microbiota contributes to the therapeutic effects of GLP-1RAs and SGLT-2i in T2DM by summarizing current clinical and preclinical findings on drug–microbiota interactions. Specifically, it evaluates the role of microbiota-derived metabolites in glycemic and metabolic regulation, and discusses the potential for incorporating microbiome profiling into individualized treatment strategies.

This review adopts a structured narrative framework designed to integrate mechanistic, translational, and clinical insights across heterogeneous study types. Relevant English-language studies were identified through PubMed, Scopus, Web of Science, Embase, and Google Scholar up to May 2025 using combinations of the terms “GLP-1 receptor agonist,” “SGLT2 inhibitor,” “gut microbiota,” “type 2 diabetes,” “renal,” and “cardiovascular.” Both preclinical and clinical investigations were considered, with an emphasis on peer-reviewed studies addressing microbiota composition, metabolite shifts, and systemic effects.

While this approach allows for comprehensive synthesis across mechanistically diverse data, it does not employ formal systematic review or meta-analytic methods. Therefore, potential selection bias cannot be excluded, and reproducibility is inherently limited compared to systematic analyses. Nonetheless, efforts were made to ensure balanced representation of both supportive and null findings. While several recent systematic and scoping reviews have summarized microbiota alterations associated with GLP-1 receptor agonists and SGLT-2 inhibitors, the present work expands on these by bridging mechanistic, translational, and clinical perspectives to delineate how gut microbiota modulation may contribute to their cardiovascular and renal benefits. In particular, this review integrates emerging human data with mechanistic evidence from preclinical studies, emphasizing shared and divergent pathways between the two drug classes. Additionally, it consolidates recent insights on combination therapy and positions the microbiota as both a potential biomarker and therapeutic co-target: topics that have been mentioned but not critically contextualized in prior reviews. Rather than claiming novelty in theme, this work provides a timely, integrative interpretation of converging findings to inform future hypothesis-driven and interventional research.

## OVERVIEW OF GUT MICROBIOTA AND METABOLIC HEALTH

Physiological activities of the gut microbiota include regulation of vitamin synthesis and absorption, bile acid metabolism, and intestinal barrier integrity [1, 4]. In addition to its local gut functions, the gut microbiota also produces bioactive metabolites, such as short-chain fatty acids (SCFAs), lipopolysaccharides (LPS), secondary bile acids, and generates precursors of uremic toxins, targeting systemic distant organs such as the liver, kidney, and cardiovascular system [5, 6]. As examples, dietary choline and carnitine are metabolized by gut microbiota to trimethylamine (TMA), which is absorbed and oxidized by the liver to the uremic toxin trimethylamine-N-oxide (TMAO) [7]. In addition, tryptophan and tyrosine are metabolized by gut microbes to indole and p-cresol, respectively, which are then metabolized by human cells to the protein-bound uremic toxins indoxyl sulfate and p-cresyl sulfate.

Gut dysbiosis is often linked with metabolic diseases such as obesity, insulin resistance, and T2DM and usually characterized by profoundly impaired microbial diversity and function [8]. Common indicators of dysbiosis are a decrease in favorable bacteria such as *Faecalibacterium prausnitzii* and *Akkermansia muciniphila* and an increase in pro-inflammatory species that weaken the gut barrier and promote systemic inflammation [9]. In particular, long-term low-grade inflammation can be triggered by LPS and is one of the major contributors of insulin resistance and endothelial dysfunction, two characteristics of the metabolic syndrome [10, 11].

The SCFAs acetate, propionate, and butyrate are among the most studied microbial metabolites; they bind to G-protein-coupled receptors and regulate energy metabolism, appetite, and glucose balance [12]. Furthermore, they also modulate epigenetic-dependent gene expression through regulation of histone acetylation [13]. Specifically, butyrate prevents the kidney downregulation of the antiaging, kidney protective and anti-inflammatory protein Klotho [14]. Low SCFA synthesis may exacerbate metabolic problems in dysbiotic conditions, even if glycemic control is improved when the microbial balance is altered by food, medication, or other interventions [3].

Altered gut microbiota homeostasis is associated with the development of cardiovascular and chronic kidney disease, in addition to their involvement in metabolic diseases. Low SCFA production may compromise kidney and tissue resilience while uremic toxins such as TMAO and others promote atherosclerosis, vascular inflammation, and kidney disease progression [2, 15]. Overall, these observations highlight the relevance of gut microbiota for overall metabolic health and suggest that treatment of T2DM and its associated complications may interact with the gut microbiota.

## GUT MICROBIOTA AND CHRONIC KIDNEY DISEASE

A recent study investigated the complex relationship between gut microbiota, uremic toxins (UTs), diet, and the progression of chronic kidney disease (CKD) in non-dialysis patients [16]. 240 CKD patients followed for 3-year were compared to healthy volunteers. Results showed that CKD patients have altered gut microbiota compared to healthy controls, with an enrichment of species that produce UT precursors. Patients with more severe CKD exhibited higher levels of UTs and a greater abundance of UT-producing species. Longitudinal analysis revealed an increase in UT-producing species over time, correlating with CKD progression. Experimental models using fecal microbiota transplantation (FMT) from CKD patients into antibiotic-treated CKD mice demonstrated a causal link: these mice experienced increased serum UT levels and exacerbated kidney fibrosis. Specific species such as *Enterocloster* and *Hungatella genera* were enriched in CKD patients and harbored genes for UT production, negatively correlating with kidney function. Conversely, *F. prausnitzii*, a protective bacterium, was depleted in severe CKD and fast CKD progressors, and its abundance was inversely correlated with UT levels and kidney fibrosis. The study also highlighted the influence of diet on gut microbiota modifications. A plant-based, low-protein diet appeared to mitigate the increase in UT-producing species over time. Increased vegetable intake and stable probiotic consumption were associated with a reduction in the “toxic species ratio,” while decreased fiber intake led to an increase. These findings suggest that dietary interventions targeting the gut microbiome could be a potential therapeutic strategy to slow CKD progression by reducing UT accumulation.

## GLP-1 RECEPTORS, GLP-1 RECEPTOR AGONISTS, AND GUT MICROBIOTA

Glucagon-like peptide-1 receptors (GLP-1Rs) are G protein-coupled receptors that are found in pancreatic β-cells, intestinal L-cells, neurons, and immune cells. When activated, they cause insulin to be released, stop glucagon release, slow down the emptying of the stomach, and increase satiety, all of which are important for keeping blood sugar levels stable and managing appetite [11, 17].

There is increasing evidence that the gastrointestinal microbiota is influenced by GLP-1 signaling in a bidirectional manner. Microbial metabolites, especially SCFAs such as butyrate and propionate, boost the body’s own GLP-1 secretion by activating G-protein-coupled free fatty acid receptors [4]. On the other hand, administrating GLP-1RAs such as liraglutide and semaglutide modulates the microbiota composition by increasing beneficial taxa such as *A. muciniphila* and *Bacteroides*, making potentially harmful bacteria less common [4].

In addition, activating GLP-1Rs helps keep the intestinal barrier strong and the immune system in balance. This is partly due to modulation of inflammatory signaling pathways and improvement of mucosal function, for example, by modulating the metabolism of bile acids [18, 19], and by increasing beneficial microbes that support gut homeostasis [4]. Thus GLP-1Rs would behave as molecular links between the health of gut microbes and the overall metabolic health of the body.

### Mechanisms of GLP-1RAs actions relevant to the gut

In the gastrointestinal system GLP-1RAs delay stomach emptying, increase satiety signals, decrease intestinal motility, and regulate bile acid metabolism [1, 5]. It is possible that GLP-1RAs may directly affect the gut environment, as the presence of GLP-1 receptors on enteric neurons, immune cells, and enteroendocrine L-cells throughout the gut suggests [5, 20]. This local gut distribution of GLP-1 receptors supports a potential bidirectional interaction between GLP-1 signaling and the gut microbiota, which could potentially contribute to the therapeutic benefits observed.

### Direct effects on microbiota composition

Preclinical and clinical studies demonstrate that GLP-1RAs modulate the gut microbiome, restoring dysbiotic patterns and encouraging bacterial diversity. Notably, liraglutide treatment was associated with an increase in beneficial taxa such *Lactobacillus, Bacteroides*, and *A. muciniphila*, as well as a decrease in the *Firmicutes* to *Bacteroidetes* ratio, a crucial dysbiosis marker in metabolic diseases [6, 10]. These changes are associated with weight reduction and better glucose tolerance, which suggests that changes in the microbiota may both reflect and encourage metabolic improvement [6].

Furthermore, by creating niches for specific bacterial populations, GLP-1RAs may influence microbial richness through altered pH profiles, bile acid modulation, and altered gut motility [5, 11]. GLP-1Ras treatment was associated with increased numbers of two types of bacteria that make SCFA, *Roseburia* and *F. prausnitzii*, in both human and animal investigations. These bacteria are linked to better blood glucose regulation, a stronger intestinal barrier, and less inflammation [9, 12].

### Microbiota-derived metabolites and pathways

Changes in the microbiota brought on by GLP-1RAs are associated with changes in key microbial metabolite patterns that may impact host metabolism. Following GLP-1RA treatment, SCFAs, such as butyrate and propionate, are elevated [9, 10]. These SCFAs are known to improve insulin sensitivity, regulate appetite and hormones such as peptide YY (PYY), and reduce systemic inflammation [6, 12]. Reduced levels of gut-derived endotoxins, such as LPS, indicate that the intestinal barrier is intact and that pro-inflammatory chemicals are not translocating as much [5, 10, 11].

There is a bidirectional interaction between GLP-1/biliary acids and the gut microbiota [21, 22]. Bacterial-derived hyodeoxycholic acid (HDCA) promotes GLP-1 secretion by upregulating the expression of the bile acid receptor Takeda G protein-coupled receptor 5/G-protein-coupled bile acid receptor 1 and downregulating the expression of the bile acid receptor farnesoid X receptor in the gut [22]. In addition, liraglutide increase the serum levels of deoxycholic acid [19] and thus, may also have an impact on bile acid signaling. Hepatic lipid metabolism, glucose regulation, and gut hormone release are all affected by these relationships [3, 11]. Together, these results indicate that gut microbiota and its metabolites may actively contribute to the systemic effects of GLP-1RA therapy, rather than serving as passive responders.

### Clinical implications and relevance of microbiota modulation by GLP-1RAs

Emerging clinical evidence also suggests that gut microbiota may play a mechanistic or modulatory role in the therapeutic efficacy of GLP-1 receptor agonists in T2DM, although findings remain heterogeneous across studies. In a pilot study involving 52 patients with T2DM treated with GLP-1RAs, 16S rRNA amplicon sequencing was used to characterize gut microbial composition and identify taxa associated with glycemic response. Responders and non-responders exhibited significantly different beta diversity profiles, with specific microbes such as *Bacteroides dorei* and *Roseburia inulinivorans* positively correlating with HbA1c reduction, while taxa including *Prevotella copri* and *Mitsuokella* spp. were negatively associated, suggesting that baseline gut microbiota may serve as a predictive biomarker for GLP-1 RA efficacy in clinical management of T2DM [23].

However, some studies have reported contrasting results. For example, in a 12-week randomized, double-blind, placebo-controlled trial (NCT01744236), 51 adults with T2DM, already receiving metformin and/or sulfonylureas, were assigned to liraglutide (1.8 mg), sitagliptin (100 mg), or placebo to assess the impact of GLP-1 receptor agonism and DPP-4 inhibition on gut microbiota composition. Despite significant improvements in glycemic control with both agents, particularly a 1.3% HbA1c reduction with liraglutide, and modest weight loss, 16S rRNA sequencing of fecal samples showed no significant changes in either alpha or beta diversity of the gut microbiota across groups. In addition, microbiota composition did not correlate with clinical outcomes, although shifts in fecal bile acid profiles (e.g. increased deoxycholic acid with liraglutide, and cholic/chenodeoxycholic/ursodeoxycholic acids with sitagliptin) were observed [24]. On the other hand, while no significant changes in microbial diversity or composition were observed after the first week of dulaglutide treatment, long-term (>48 weeks) administration led to a significant reduction in microbial abundance (as indicated by Chao1 and Ace indices) and a distinct shift in community structure (beta diversity), without changes in alpha diversity in a longitudinal clinical study of 41, newly diagnosed, treatment-naïve T2DM patients. Notably, key metabolic markers, including fasting glucose, HbA1c, BMI, and fasting C-peptide, were significantly correlated with the abundance of specific bacterial genera such as *Bifidobacterium, Bacteroides, Ruminococcus*, and *Blautia* [25]. Overall, these findings might suggest that the metabolic benefits of liraglutide, at least in the short term, may occur independently of detectable changes in gut microbial diversity or composition. Meanwhile, dulaglutide, for example, may exert its metabolic benefits in part through long-term stepwise modulation of gut microbial composition, highlighting a potential bidirectional relationship between GLP-1 receptor agonism and the intestinal microbiome in T2DM treatment.

In this context, another consideration is that while GLP-1RAs analogs influence the composition and diversity of the gut microbiota in both animal and human models, effects might potentially vary significantly by drug type, population characteristics, and treatment duration. A recent systematic review analyzed 38 preclinical and clinical studies to assess the impact of GLP-1RAs, including liraglutide, exenatide, dulaglutide, and semaglutide, on gut microbiota composition, diversity, and metabolic relevance, showed that while liraglutide consistently promoted the enrichment of metabolically favorable genera (e.g. *Akkermansia, Bacteroides*), dulaglutide was associated with increases in *Ruminococcus* and *Akkermansia*, while semaglutide produced mixed outcomes, enhancing *A. muciniphila* abundance but reducing microbial diversity, therefore suggesting that GLP-1RAs exert compound- and context-specific effects on the microbiome that may depend on host factors, treatment duration, and baseline microbial composition [26].

Microbiota effects may also contribute to the extra-glycemic benefits of cardiovascular and kidney protection, and anti-inflammatory activity. For instance, improved endothelial function and a slower rate of atherosclerosis progression are linked to increased abundance of *A. muciniphila* [10, 26]. SCFA enrichment is linked to fewer oxidative stress markers and more favorable glycemic variability [3]. Thus, shifting gut microbiota could be a sign of therapy responsiveness and a possible therapeutic enhancer.

Potential future applications in microbiome-personalized diabetes care may be supported by a few early studies that also indicate the baseline microbiota composition may predict the extent of the metabolic response to GLP-1RAs, suggesting that treatment of diabetes in a microbiome-specific manner should be explored [3, 12]. However, more extensive and continuous human studies are still required to validate these findings.

## SGLT-2 INHIBITORS AND GUT MICROBIOTA

SGLT-2is are prescribed to treat T2DM but are also considered first line therapy for heart failure and CKD [27].

### Mechanisms of SGLT-2is beyond glycosuria

SGLT-2is, including dapagliflozin, empagliflozin, and canagliflozin, act primarily by inhibiting renal glucose reabsorption in the proximal tubules, thus promoting glycosuria. However, in addition to decreasing serum glucose, they have a wide range of metabolic effects. Notably, SGLT-2is induce natriuresis and osmotic diuresis, causing a contraction in plasma volume, reduction in blood pressure, and improvement in cardiac preload and afterload [6, 28]. They also decrease glomerular hyperfiltration, increase hemoglobin levels and promote the production of the kidney antiaging protein Klotho, which delays cardiovascular aging [29]. These effects are believed to contribute to the observed cardiovascular and renal protective outcomes in major clinical trials.

SGLT-2is promote a metabolic transition toward ketogenesis by enhancing hepatic lipid oxidation and increasing circulating ketone bodies, which serve as efficient energy substrates for the kidneys and heart [30]. These ketones, especially β-hydroxybutyrate, may enhance mitochondrial efficiency and possess anti-inflammatory characteristics. Furthermore, SGLT-2is promote uricosuria, which lowers the blood uric acid levels. This action may be significant because hyperuricemia is linked to endothelial dysfunction and cardiovascular disease [31]. Together, these systemic effects create a metabolic environment that may positively influence the gut microbial environment. Despite increased uricosuria, they decrease the risk of urolithiasis in part through increased urinary citrate [32].

### Evidence of microbiota modulation by SGLT-2is

Recent research indicates that SGLT-2is directly modifies the composition and function of the gut ecosystem. Preclinical and human research studies have documented an increased abundance of bacteria producing SCFA such as *F. prausnitzii, Roseburia*, and *Bifidobacterium* following SGLT-2i administration [1, 33]. These bacteria are linked to better gut-barrier function and anti-inflammatory effects. Also, SGLT-2is have been associated with a lower *Firmicutes/Bacteroidetes* ratio and a lower number of pathobionts such *Enterobacteriaceae*, which suggests that the body is moving toward a more eubiotic state [34].

Furthermore, SGLT-2i have been linked to elevated SCFA levels in the colon, specifically butyrate and acetate, which are known to affect insulin sensitivity, glucose homeostasis, and appetite control and protect from kidney and cardiovascular disease [14, 35]. Reductions in systemic LPS levels, which serve as a marker of endotoxemia and gut-barrier dysfunction, support these metabolic benefits [6]. SGLT-2is may affect microbiota through modifications in intestinal pH, bile acid composition, and local nutrition availability, all of which influence microbial habitats.

### Contribution to renal and cardiovascular outcomes

The gut microbiota may have a mechanistic impact in improving the renoprotective and cardioprotective benefits of SGLT-2is. Microbial profiles associated with improvement in SCFAs are connected to diminished systemic irritation, improved endothelial work, and decreased oxidative stress, all critical for vascular and renal wellbeing [2, 33]. In murine models, changes in microbiota initiated by SGLT-2is were connected to diminished renal fibrosis and decreased generation of pro-inflammatory cytokines. Research shows that modifications in microbiota can be related with protection from kidney injury and improved kidney function, as shown by lower albuminuria levels and stability of glomerular filtration rate [1].

Alongside improvements in fasting glucose and weight maintenance, dapagliflozin was associated with a progressively greater shift in gut microbiota composition over time, particularly influencing taxa such as *Lactobacillus* and *Muribaculaceae* in a diabetic kidney disease mice model [36]. A previous preclinical mice study investigated whether the therapeutic benefits of empagliflozin in T2DM-related diabetic nephropathy were mediated via modulation of the gut microbiota [37] Using a high-fat diet and streptozotocin (HFD/STZ)-induced mouse model of T2DM-associated diabetic nephropathy, male mice were randomized to receive empagliflozin or vehicle for four weeks. Empagliflozin promoted renal protection by significantly reducing blood glucose, urine albumin-to-creatinine ratio, and renal histopathologic injury while restoring gut microbiota diversity and epithelial barrier integrity. 16S rRNA sequencing revealed reductions in LPS-producing bacteria (e.g. *Oscillibacter*) and increases in SCFA-producing taxa (e.g. *Bacteroides, Odoribacter*), accompanied by elevated fecal SCFA levels and reduced fecal and serum LPS concentrations. Meanwhile, antibiotic-treated cohorts showed diminished renal and intestinal effects of empagliflozin, supporting a microbiota-dependent mechanism [37].

There is also some clinical evidence that empagliflozin’s cardiometabolic benefits in early T2DM may be partly mediated through gut microbiota modulation and systemic metabolic remodeling. For example, empagliflozin treatment uniquely improved cardiovascular disease risk profiles, increased microbiota richness and SCFA-producing taxa, and shifted circulating metabolites toward a more favorable cardiometabolic profile compared to metformin in a randomized clinical trial of 76 treatment-naïve adults with T2DM and at least one cardiovascular disease risk factor [38]. Similarly, reduced abundance of *Fusobacterium* following dapagliflozin treatment was associated with improved glycemic, lipid, inflammatory, and endothelial parameters, as observed in an 8-week, single-arm clinical trial involving 12 patients with T2DM treated with metformin [39].

### Gut microbiota modulation by SGLT-2 inhibitors and its metabolic impact: evidence from human studies

Current clinical evidence from human studies exploring the microbiota-related metabolic, clinical, or prognostic effects of SGLT-2is remains limited. In a double-blind, randomized trial of 44 metformin-treated patients with T2DM, neither dapagliflozin nor gliclazide significantly altered gut microbiome composition over 12 weeks, despite exhibiting divergent metabolic effects such as reduced weight and fasting insulin with dapagliflozin and increased insulin with gliclazide [40]. Both agents improved glycemic control comparably, but the absence of changes in microbial diversity or taxa suggests their metabolic actions are not mediated via alterations in the fecal microbiota.

In addition, SGLT-2i use in pre-dialysis CKD patients was associated with reduced circulating levels of protein-bound UTs of bacterial origin (indoxyl sulfate and p-cresyl sulfate), alongside distinct shifts in gut microbial composition, particularly enrichment of *Bacteroides stercoris* and *Bacteroides coprocola*, and changes in microbial functions linked to protein and carbohydrate metabolism in a matched case-control study of 60 CKD patients (SGLT-2i treatment, *n* = 30; untreated, *n* = 30) and 30 non-CKD controls. Although serum SCFA levels were lower in SGLT-2i treated patients, the study also identified gut taxa–metabolite correlations implicating a host–microbe–metabolite axis in CKD [41].

## COMBINED USE OF GLP-1RAs AND SGLT-2IS: ANY SYNERGISTIC EFFECTS?

There is interest in combination treatment utilizing GLP-1RAs and SGLT-2is due to their synergistic impacts on improving metabolic and cardiovascular results. This approach has been shown to improve glycemic control, encourage weight loss, and decrease cardiovascular issues over time [5, 42]. Insufficient information exists with respect to the impact on the gut microbiome of these cumulative effects.

In preliminary findings from preclinical studies, concurrent therapy with GLP-1RAs and SGLT-2is may change intestinal microbial biology in distinctive or synergistic ways. Combinatorial models have illustrated an increment in *A. muciniphila* and SCFA-producing taxa, alongside raised SCFA concentrations and diminished intestinal permeability [1, 2]. These microbial changes are related with improved metabolic and inflammatory profiles, suggesting a potential contribution of microbiota to beneficial clinical effects.

However, human data remain limited. Therefore, it remains unclear whether the impacts of GLP-1RAs and SGLT-2is on gut microbiota are additive, redundant, or interactive. Clinical studies exploring microbiological endpoints, metagenomic analysis, and functional tests are needed.

## INDIRECT PATHWAYS AND EMERGING CONCEPTS

Emerging concepts include the influence of the gut microbiota on patient responsiveness to SGLT-2is and GLP-1RAs, which may potentially allow patient selection based on gut microbiota patterns, targeting the microbiota to optimize patterns associated with therapeutic responsiveness and, in essence, a path toward precision and personalized medicine.

### Microbiota as biomarkers of drug responsiveness

The glucose lowering response to SGLT-2is and GLP-1RAs may be anticipated by the nature of the gut microbiota, according to recent studies. *A. muciniphila* and *F. prausnitzii* have been associated to improved glycemic response in people taking GLP-1RAs [26, 43]. These associations support a novel hypothesis that proposes that gut microbial patterns may serve as predictive biomarkers of therapeutic efficacy. More broadly available metagenomic sequencing may allow the use of machine learning to stratify patients based on baseline gut microbiome composition and expected response to therapy. Microbiome-informed patient selection and customized treatment plans may result from this research.

### Microbiota as a therapeutic target

In addition to being a biomarker, the gut microbiota might also become a modifiable component that can improve the therapeutic impacts of SGLT-2is and GLP-1RAs. Intervention that promote a healthy microbiota, such as a heathy diet rich in fiber, or probiotics, prebiotics, and even postbiotics may improve gut-barrier integrity, boost the synthesis of SCFAs, and potentially potentiate the benefit of GLP-1RAs [5, 6, 44]. FMT may also be used. It is already used to treat severe diarrhea caused by *Clostridium difficile* (https://www.nice.org.uk/guidance/mtg71/chapter/1-Recommendations). Recent trials have confirmed the feasibility of using pills (dubbed “poo pills” by the lay press) for FMT to modify the gut microbiota [45]. There is less information on SGLT-2is and the influence of the gut microbiota [33]. Combination techniques that use microbiome-directed therapeutics to supplement pharmaceutical treatment become possible as a result.

In more advanced renal disease, the potential changes in microbiota during the progression of kidney disease [16] may alter the effects of either drug or their combination. Alternatively, SGLT-2is and GLP-1RAs may correct these microbiota alterations and possibly induce improvements in metabolic profile seen in more advanced kidney disease, e.g. below an eGFR of 30 ml/min [16, 46]. These questions should certainly be addressed in future studies.

### Personalized medicine

An emerging area of diabetes management is precision and personalized diabetes treatment based on gut microbiota analysis. Individualized therapy regimens based on microbiome profiles might be possible with a better understanding of host–microbe interactions. Depending on their microbial composition, some patients may benefit more from SGLT-2is, whereas others with low *Akkermansia* abundance may theoretically be candidates for GLP-1RA therapy [26, 43].

While microbiota-informed personalization is an appealing concept, translation into clinical practice remains preliminary. Current evidence primarily supports associations rather than causality, and predictive microbial signatures require validation in independent, large-scale cohorts before guiding therapy selection. Although developing frameworks propose integrating multi-omics data—including clinical indicators, and metabolomic and microbial profiles—to design personalized regimens, these approaches remain experimental. Robust validation in prospective clinical trials is essential before microbiota-informed therapy can be incorporated into standard care [5, 33].

## LIMITATIONS OF CURRENT EVIDENCE AND GAPS IN KNOWLEDGE

Despite the increasing information on gut microbiota as a modulator of antidiabetic drug effects, there are still systematic and conceptual gaps in the data available. Preclinical models are often used. However, differences between human and murine gut microbiota as well as in drug pharmacokinetics and metabolism mean that human confirmation is required [5, 43].

Due to different research designs, including drug type, dose, duration, patient population, and basic microbiota variation, it is difficult to compare results or achieve consistent conclusions from human studies. The gut microbial landscape can be significantly altered by confounding factors such the role of kidney failure per se [16], the large variations of dietary patterns worldwide, including animal or vegetable sources of food, antibiotic usage, and probiotic supplements, but few studies take these factors into account and many lack adequate controls [1, 6]. Furthermore, 16S rRNA sequencing, which provides taxonomic but not functional resolution, is sometimes the only method available for metagenomic and metabolomic investigations [6]. This limits our understanding of how microbial changes modulate the host response.

Although a formal quality assessment of individual studies was not conducted, methodological heterogeneity, sample size, and design limitations were critically appraised narratively to provide a balanced interpretation. In addition, publication bias may skew the available evidence toward positive or mechanistically appealing results, whereas neutral or negative findings remain underreported. Extrapolating from animal studies should also be done cautiously, as species-specific differences in microbiota composition, metabolism, and drug pharmacokinetics can markedly affect translational relevance. Furthermore, most current studies are associative rather than causal; without proof that the microbiota is a mediator, it remains uncertain whether observed microbial changes drive or merely accompany clinical effects. Only a few investigations have incorporated microbiota-modulating interventions (e.g. prebiotics or FMT) to test causality directly. Furthermore, evidence on combined GLP-1RA and SGLT-2i therapy in the context of microbiota modulation remains scarce, making it difficult to determine whether their effects are redundant, additive, or synergistic [2, 11].

Last, tailored techniques that include microbial profiles in clinical decision-making are yet theoretical, despite some research suggesting that the baseline microbiome may affect therapy response. To prove causation, predictive microbial signatures should be identified and validated in independent cohorts. Moreover, it should be determined whether altering the microbiome can improve the effectiveness of GLP-1RA and SGLT-2i treatment. Longitudinal, multi-omics studies with well-matched control arms are required [12, 43].

## ONGOING TRIALS

Currently, a limited number of clinical trials are exploring how SGLT-2is may influence gut microbiota composition. An ongoing interventional, single-arm, open-label trial (Dapagut study, NCT05965440) is evaluating the effects of dapagliflozin on gut microbiota composition and microbiota-derived metabolites over 12 weeks in non-diabetic adults with CKD. Gut microbial alpha and beta diversity will be assessed using 16S rRNA sequencing. Blood, urine, stool, and dietary data will be collected to investigate potential links between microbiota modulation, uremic toxin profiles, and renal outcomes. This study addresses the hypothesis that dapagliflozin’s renal benefits may be partially mediated through gut microbiota pathways in the absence of diabetes.

To date, no ongoing clinical trials have evaluated the combined effects of GLP-1RAs and SGLT-2 inhibitors on gut microbiota, representing an important gap for future translational research. While preliminary findings are promising, existing trials remain limited in size and scope, emphasizing the need for standardized, longitudinal human studies integrating multi-omics analyses to establish causality.

## CONCLUSION AND FUTURE PERSPECTIVES

GLP-1RAs and SGLT-2is have been utilized in recent years to improve metabolic control in people with T2DM and to improve cardiovascular and kidney outcomes in people with or without diabetes. An increasing amount of research suggests that gut microbiota may interact with these interventions and potentially play a significant mediating role in their beneficial systemic effects, while the exact mechanisms are still unclear.

There is at least preclinical evidence that GLP-1RAs and SGLT-2is may modulate the composition and activity of gut microorganisms to produce metabolites such as bile acids, SCFAs, and endotoxins. Such changes may also impact insulin sensitivity, organ function, and systemic inflammation, suggesting a new microbiota-mediated axis in the treatment of T2DM.

Despite promising preclinical and early clinical outcomes, much remains to be discovered. Most available microbiome research lacks a common gold-standard methodology, is associative, and uses small cohorts or animal models. To ascertain and confirm the impact of drug-induced microbial alterations on clinical outcomes, extensive, meticulously designed human research is required.

While mechanistic links between gut microbiota and the extra-glycemic effects of GLP-1RAs and SGLT-2is are biologically plausible, the current body of evidence remains largely associative and hypothesis-generating. Translation to clinical outcomes requires caution, as most studies rely on small cohorts, short-term interventions, and surrogate markers rather than hard endpoints. Therefore, the discussion of therapeutic implications in this review should be interpreted as conceptual rather than confirmatory.

**Figure 1:**
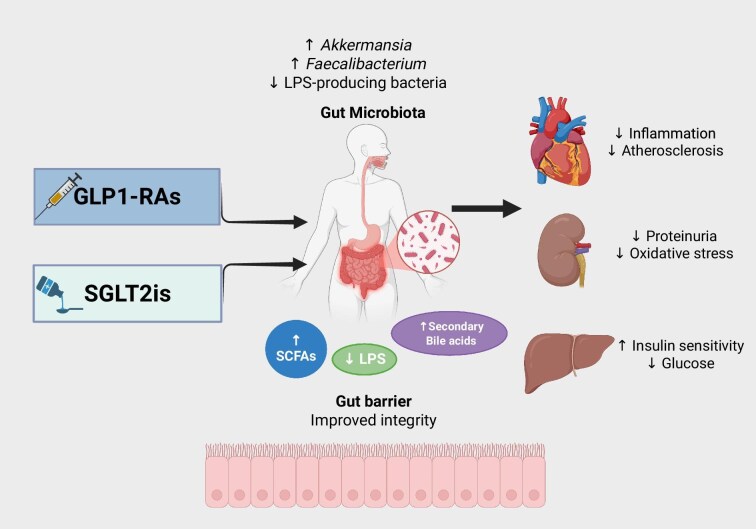
Mechanistic pathways through which GLP1-Ras and SGLT-2is may modulate gut microbiota and potentially contribute to systemic, metabolic, cardiovascular, and renal benefits. Both GLP-1RAs and SGLT-2is beneficially modulate gut microbiota composition and its metabolites. These agents are associated with increased abundance of *Akkermansia* and *Faecalibacterium* and reduced LPS-producing bacteria, resulting in higher production of SCFAs and secondary bile acids. These changes improve gut-barrier integrity and reduce endotoxemia, oxidative stress, and inflammation, ultimately contributing to enhanced insulin sensitivity, decreased glucose levels, reduced proteinuria, and protection against atherosclerosis. Together, these pathways provide a mechanistic link between gut microbiota modulation and the systemic cardio-renal-metabolic benefits observed with GLP-1RAs and SGLT-2is.

Future clinical decision-making may employ microbiota characterization to provide more individualized diabetes care. Grouping patients according to their gut microbial profiles may predict their response to SGLT-2is or GLP-1RAs and suggest microbiota-modifying interventions such as FMT; dietary modifications; or pre-, pro-, or postbiotics to optimize therapeutic results. Combination therapy approaches that target human and microbial pathways may also be revolutionary in the treatment of metabolic illnesses (Fig. 1, Tables 1 and 2).

**Table 1:** Summary of GLP-1RA and SGLT-2i effects on gut microbiota, key microbial metabolites, and associated systemic outcomes.

Medication class	Context	Microbial shifts	Key metabolites affected	Clinical effects mediated
GLP-1RAs	PreclinicalClinical	↑ *Akkermansia muciniphila* ↑ *Faecalibacterium prausnitzii* ↑ *Roseburia* ↑ *Bacteroides* ↓ *Firmicutes/Bacteroidetes* ratio ↑ microbial diversity	↑ SCFAs (butyrate, propionate), ↓ LPS, ↑ secondary bile acids	↓ Inflammation, ↑ insulin sensitivity, ↓ atherosclerosis, ↓ gut permeability, improved glycemic control
SGLT-2i	PreclinicalClinical	↑ *Bacteroidetes* ↓ *Proteobacteria* ↑ microbial diversity (less consistent than for GLP-1RAs)	↑ SCFAs ↓ UTs (e.g. TMAO, indoxyl sulfate)	↓ renal oxidative stress, ↓ proteinuria, improved lipid/glucose metabolism
Combined	Preclinical	Additive effects on microbial diversity and metabolite enrichment	Possibly synergistic ↑ SCFAs and ↓ LPS and TMAO	Enhanced cardiometabolic protection, improved glycemic control, and renal outcomes; further research needed to confirm mechanisms
	Clinical	No data	No data	No data

Abbreviation: F/B ratio (*Firmicutes* to *Bacteroidetes* ratio)

**Table 2:** Existing knowledge, unknowns, and research recommendations on microbial impact of GLP-1RA and SGLT-2i.

	Existing knowledge	Unknowns	Research recommendations
GLP-1RA	Increased *Akkermansia, Faecalibacterium*, SCFA producers; reduced LPS and systemic inflammation. Effects observed in both mice and humans.	Long-term microbial stability; causality between microbial changes and outcomes. Microbiota modulation of drug pharmacokinetics.	Conduct large-scale, longitudinal human studies; integrate shotgun metagenomics and metabolomics; test microbiota-targeting co-interventions (e.g. prebiotics).
SGLT-2i	Mild increase in diversity; decreased UTs and *Proteobacteria*. Suggested renal and metabolic benefits linked to microbiota.	Mechanistic pathways linking microbial changes to renal protection unclear; impact on bile acid pathways underexplored; species-level resolution is limited.	Incorporate microbiome analysis in SGLT-2i trials; examine bile acid signaling and SCFA profiles; explore combined effect with GLP-1RA in microbiota-focused models.

In conclusion, using gut flora with antidiabetic medications may be a compelling way to enhance the results of T2DM. Precision medicine informed by the microbiome may result from defining these intricate interactions.

## Data Availability

No new data were generated or analyzed in support of this research.
